# Comparative effects of intermittent fasting and calorie restriction on cardiovascular health in adults with overweight or obesity

**DOI:** 10.1038/s41598-025-32673-9

**Published:** 2025-12-15

**Authors:** Niloufar Abdollahpour, Najmeh Seifi, Mina Nosrati, Habibollah Esmaily, Ali Mottaghi Moghaddam Shahri, Gordon A. Ferns, Maryam Alinezhad-Namaghi, Majid Ghayour-Mobarhan

**Affiliations:** 1 Department of Nutrition, Faculty of Medicine, Mashhad University of Medical Sciences, Mashhad, 99199- 91766 Iran; 2 Department of Biostatistics, School of Health, Mashhad University of Medical Sciences, Mashhad, Iran; 3 Social Determinants of Health Research Center, Mashhad University of Medical Sciences, Mashhad, Iran; 4 Division of Medical Education, Sussex Medical School, Brighton &amp, Falmer, Brighton, Sussex UK; 5 Transplant Research Center, Clinical Research Institute, Mashhad University of Medical Sciences, Mashhad, Iran; 6 Metabolic Syndrome Research Center, Mashhad University of Medical Sciences, Mashhad, 99199- 91766 Iran

**Keywords:** Obesity, Intermittent fasting, Calorie restriction, Heart disease risk factors, Blood pressure, Cardiology, Diseases, Endocrinology, Health care, Medical research, Risk factors

## Abstract

Cardiovascular disease (CVD) risk factors are a significant global health concern. Previous studies have demonstrated that lifestyle-based strategies such as 5:2 intermittent fasting (IF) and calorie restriction (CR) may improve blood pressure, lipid profiles, glycemic control, and cardiovascular risk scores. However, comparative evidence on their effects in real-world settings remains limited. This study aimed to compare the effects of 5:2 IF and CR on cardiovascular risk factors in overweight and obese adults. This longitudinal cohort study used data from the Iranian National Obesity Registry (IRNOR). A total of 82 participants were included (40 in the 5:2 IF group and 42 in the CR group). Participants followed either a 5:2 IF (500–600 kcal on fasting days, isocaloric on other days) or a daily CR approach (500–1000 kcal deficit). Cardiovascular risk factors including blood pressure indices, lipid profile components, glycemic markers, and CVD risk scores were compared between groups over three months. The mean age of participants was 35.55 ± 12.18 years (70.7% female). At the end of week 12, mean arterial pressure and rate-pressure product significantly decreased in both groups compared to that of the baseline (P < 0.05). The 5:2 IF group also experienced a significant decrease in serum triglyceride levels (P = 0.04). Additionally, at the end of the study, systolic blood pressure (123.78 ± 9.95 vs. 127.62 ± 12.16), pulse pressure (41.53 ± 6.76 vs. 46.51 ± 9.36), and the 30-year Framingham cardiovascular risk score for full CVD (19.17 ± 14.13 vs. 21.53 ± 15.10) and for hard CVD (10.31 ± 8.51 vs. 12.00 ± 9.88) were significantly lower in the 5:2 IF group compared to the amounts for the CR group (P < 0.05). However, no significant differences were observed in other metabolic parameters between the two groups (P > 0.05). Intermittent fasting regimen, particularly the 5:2 IF, may be associated with greater improvement in systolic blood pressure, pulse pressure, and the 30-year cardiovascular risk score over three months. Other metabolic outcomes were similar between the groups. Further studies are needed to confirm these findings.

**Supplementary Information:**

The online version contains supplementary material available at 10.1038/s41598-025-32673-9.

## Introduction

Cardiovascular disease (CVD) is a crucial global health concern, accounting for approximately 19.8 million global deaths each year^1^. In addition to genetic predisposition and aging, modifiable lifestyle factors, particularly excess weight and metabolic dysfunction, play a key role in the etiology of this disease. The growing prevalence of obesity and its associated conditions such as hypertension, dyslipidemia, and insulin resistance have significantly intensified the global CVD burden^2^. According to the World Health Organization, in the year 2022, 39% of adults aged 18 years and older were overweight or obese^3^. Similarly, recent national data show that almost one-third of Iranian adults are either overweight or obese^4^. These figures indicate that preventive weight management strategies are urgently needed to mitigate the associated health risks.

In response to the growing prevalence of metabolic syndrome and its cardiovascular implications, a range of dietary approaches have been proposed to address weight-related metabolic dysfunction^5^. From these approaches, IF, which includes several patterns such as alternate-day fasting (ADF), time-restricted feeding (TRF), and the 5:2 IF, has emerged as a promising strategy^6^. The 5:2 IF involves restricting calorie intake for two days per week, while allowing for normal eating patterns on the remaining five days. This convenient flexibility has made it a popular option for those who struggle with adopting daily calorie restriction^7^. Studies over recent decades have examined the effects of IF dietary approaches on various health indicators such as waist circumference, weight, blood pressure, lipid profile, and blood glucose levels^8,9^. Initial evidence from all this bulk of research suggests that the 5:2 IF is associated with losing weight and improving metabolic health^10^. However, there are also some studies which have reported that IF regimens may have limited effects on weight loss without controlled food intake^11^. Furthermore, findings on the effects of IF on glucose and lipid metabolism have been inconsistent across some studies^12,13^.

From a mechanistic perspective, the 5:2 IF may improve cardiovascular health through several biological pathways. This is because during fasting periods, the body shifts its primary energy source from glucose to lipids and ketones, leading to improved insulin sensitivity^14^. Additionally, IF has been associated with reduced systemic inflammation and favorable changes in adipokine profiles, such as lower leptin and higher adiponectin levels, which are linked to better vascular function and reduced atherosclerotic risk^15^. Fasting also appears to induce neuroendocrine changes, including enhanced parasympathetic activity and diminished sympathetic output, contributing to lower resting heart rate and blood pressure^16^. These physiological adaptations, along with improved endothelial function support the proposed cardiovascular benefits of IF.

Despite promising data, most existing studies were conducted under controlled conditions, limiting generalizability to real-world settings^10^. To better understand the practical effectiveness of IF, registry-based research offers a complementary approach by capturing health outcomes within routine clinical care^17^. The Iranian National Obesity Registry (IRNOR), a nationwide database of patients enrolled in multidisciplinary obesity clinics, provides a unique opportunity to evaluate the comparative cardiometabolic effects of 5:2 IF and daily CR in everyday clinical practice^18^. In contrast to earlier studies that primarily focused on conventional metabolic indices, the present study incorporated more comprehensive cardiovascular risk scores, such as the 30-year Framingham risk score and atherogenic indices to provide a novel insight for cardiovascular risk assessment. Accordingly, this study was conducted within the framework of the IRNOR to compare the effects of 5:2 IF and CR on cardiovascular risk factors in overweight and obese Iranian adults.

## Methods

### Study design and participants

This retrospective cohort study analyzed data from the electronic database of the IRNOR, which systematically records longitudinal data from adults receiving obesity treatment across clinical settings, thereby reflecting real-world clinical practice conditions. In these registry-based studies, treatments are recorded as they routinely occur in clinical practice.

In the present study, participants were categorized into the 5:2 IF or CR groups according to the dietary regimen determined by the clinical nutritionist based on standardized IRNOR protocols. These protocols apply consistent clinical criteria, including metabolic profile, comorbidities, and readiness for lifestyle modification. No direct interventions were conducted by the research team.

We included participants aged 18–65 with a body mass index (BMI) of 25 or higher. Only those who had been followed for at least three months were included in the study. Individuals who were breastfeeding or those who underwent additional weight loss interventions such as drug therapy or used body contouring procedures were excluded from the analysis. In addition, participants with conditions affecting metabolic regulation such as untreated thyroid disorders, Cushing’s syndrome, chronic kidney and liver disease or those under treatment known to interfere with energy balance, including those using systemic corticosteroids, antipsychotics, or chemotherapy, were not included. Moreover, individuals who were identified as non-adherent to the prescribed diet plan or switched dietary strategies during the three-month follow-up period were also excluded.

## Objectives and hypotheses

The primary objective of this study was to compare the effects of a 5:2 IF and CR regimens on metabolic profile in overweight and obese adults. The primary hypothesis was that participants in the 5:2 IF group would show a greater reduction in metabolic profile after 12 weeks compared with those following CR. Secondary objectives were to compare the effects of the two dietary strategies on cardiovascular risk scores.

## Sample size

The sample size calculation for each group was based on TG levels from a previous study^19^, using a formula for comparing proportions of a qualitative attribute in independent statistical populations. Based on a 5% alpha error and a 20% beta error, the required sample size was calculated to be 21 participants per study group.

$$n=\frac{(Z_{1-\frac{\alpha}{2}}+Z_{1-\beta})^{2}(SD^{2}_{1}+SD^{2}_{2})}{(Mean_{1}-Mean_{2})^{2}}$$

As the number of eligible participants was greater than the minimum required, all were included in the analysis to improve the study’s statistical power and accuracy.

## Dietary approaches

All diet plans were designed by a trained dietitian to ensure they met nutritional requirements. Moreover, each participant received dietary education from a trained dietitian to ascertain that they adhered to their prescribed diet plans. In order to estimate daily energy intake, the basal metabolic rate (BMR) was calculated employing Harris-Benedict equation and an activity factor was applied on this value to estimate total energy expenditure depending on each subject’s activity level^20,21^. In accordance with IRNOR protocol, female participants in the 5:2 IF group consumed 500 kcal, while the males received 600 kcal on two non-consecutive fasting days each week. These meals were composed of approximately 40% carbohydrates, 30% fats, and 30% proteins. Participants in both groups were advised to maintain proper hydration (at least 2 L/day). They were also permitted to consume non-caloric beverages, including water, black coffee, and unsweetened tea on fasting days to reduce the risk of dehydration. On the remaining five non-fasting days, both men and women followed an isocaloric diet that met their total energy requirements, with a macronutrient distribution of 50–55% carbohydrates, 25–30% fats, and 15–20% proteins. The CR group followed a daily energy-restricted diet with a 500–1000 kcal deficit below estimated energy needs. To maintain consistency across groups, the CR participants’ macronutrient composition was aligned with that of the 5:2 IF group’s non-fasting days (50–55% carbohydrates, 25–30% fats, and 15–20% proteins).

## Follow-up

Throughout the study, participants received weekly follow-up support and motivation from a trained dietitian via phone call. Participants’ compliance with the prescribed diets was monitored weekly using an extra food log, which recorded any additional food intake beyond their prescribed daily energy goal, in accordance with IRNOR protocol. Compliance was defined as following the prescribed diet on at least 80% of the recorded study days. In line with previous studies, this threshold was considered indicative of high compliance^22^. Only participants who maintained this level of compliance throughout the 3-month follow-up period were included in the final analysis. Additionally, body composition data was collected monthly during clinic visits. Each participant followed a specific diet that was reviewed and adjusted monthly based on changes in their weight or body composition.

### Demographic data and anthropometric measurements

Demographic information of the participants, including age, gender, marital status, and medical history was collected using a checklist. To ensure accuracy and precision, anthropometric measurements, such as height, weight, and waist circumference, were obtained using standardized procedures and equipment^23^. Height was measured using a digital standing stadiometer (InBody BSM370) with a precision of 0.1 cm. Body weight and composition were measured using a bioelectrical impedance analysis (BIA) device (InBody 770; Biospace Inc., Seoul, Korea). To ensure accurate BIA measurements, participants adhered to standard pre-test guidelines, including removing metal objects, fasting for at least 4 h, abstaining from caffeine, smoking, and alcohol, maintaining normal hydration, and avoiding vigorous physical activity for 12 h prior to assessment^24^.

## Blood pressure

Blood pressure indices were measured using the InBody BPBIO320 device, which utilizes the oscillometric method to provide highly accurate measurements (± 2 mmHg). The measured indices included systolic blood pressure (SBP), diastolic blood pressure (DBP), pulse rate (PR), pulse pressure (PP), mean arterial pressure (MAP), and rate pressure product (RPP).

## Laboratory tests

Blood tests conducted on patients during each visit were recorded following the IRNOR laboratory data recording protocol. To maintain accuracy and precision, only data from patients with both initial and final blood test records were used for analysis. Participants were referred to the same laboratory to eliminate potential variations. The laboratory tests included fasting plasma glucose (FPG), hemoglobin A1c (HbA1c), lipid profile (total cholesterol, HDL-C, LDL-C, and TG), alanine transaminase (ALT), aspartate aminotransferase (AST), urea, creatinine, uric acid, and thyroid stimulating hormone (TSH).

### 30-year cardiovascular disease risk assessment

The Framingham Heart Study model was used to estimate each individual’s 30-year cardiovascular disease risk (CVDRisk30y)^25^. Age, gender, systolic pressure, hypertension treatment, diabetes, and smoking status were included in both the BMI and lipid-based models. Additionally, the BMI-based model required BMI, while the lipid-based model required total cholesterol and HDL-C. The Framingham risk scores encompass a variety of cardiovascular diseases, including complete cardiovascular diseases (transient ischemic attacks, coronary insufficiency, angina, and congestive heart failure) and severe cardiovascular diseases (death from coronary heart disease, myocardial infarction, as well as fatal and non-fatal strokes)^26^.

Additional cardiovascular disease risk scores were calculated using the following formulas:

$${\rm Non-HDL cholesterol = Total cholesterol - HDL-C}$$

.

$${\rm Coronary risk index = Total cholesterol / HDL-C}$$

$${\rm TG: HDL-C ratio =TG/HDL-C}$$

$${\rm Atherogenic \:index =LDL-C/HDL-C}$$

$${\rm Atherogenic \:index \:of \:plasma =}\:{\mathrm{log}}_{10}\frac{\mathrm{T}\mathrm{G}}{\mathrm{H}\mathrm{D}\mathrm{L}-\mathrm{C}}$$

These metrics provided additional insights into the cardiovascular health of the participants.

#### Physical activity

Physical activity of the participants were assessed using the International Physical Activity Questionnaire-Short Form (IPAQ-SF)^27^. This questionnaire has been validated for the Persian language and is a reliable tool for the Iranian population aged 18 to 65^28,29^. The IPAQ-SF collects information on the frequency, duration, and intensity of physical activities performed in the previous week, including walking as well as moderate and vigorous activities. This data was utilized to evaluate the potential impact of physical activity on weight loss and cardiovascular health.

In accordance with the IRNOR protocol, all anthropometric measurements, blood pressure indices, laboratory tests, cardiovascular disease risk scores, physical activity and dietary intake (assessed via 3-day 24-hour dietary recalls) were routinely recorded at both baseline and the final stage of follow-up. This data was used in the present study to evaluate changes over time.

#### Statistical analysis

Qualitative data were analysed using frequencies and percentages. The Kolmogorov-Smirnov test was used to assess data normality. Quantitative data were reported as either mean and standard deviation (if normally distributed) or median and interquartile range (if not normally distributed). To compare quantitative variables between the two groups, either the student’s t-test or Mann-Whitney test was used, while the chi-square test was used for qualitative variables. To assess within-group changes before and after evaluation, paired t-tests or the Wilcoxon signed-rank test were employed. To compare the changes between the two groups, baseline values of each variable, age, sex, and BMI were adjusted, using analysis of covariance (ANCOVA). All statistical analyses were conducted using SPSS version 25, with a significance level set at 0.05.

#### Ethics

The study was conducted in accordance with the Declaration of Helsinki and approved by the Mashhad University of Medical Sciences Ethics Committee (IR.MUMS.MEDICAL.REC.1402.333). Informed consent was obtained from all participants before registry enrollment.

## Results

**Fig. 1:**
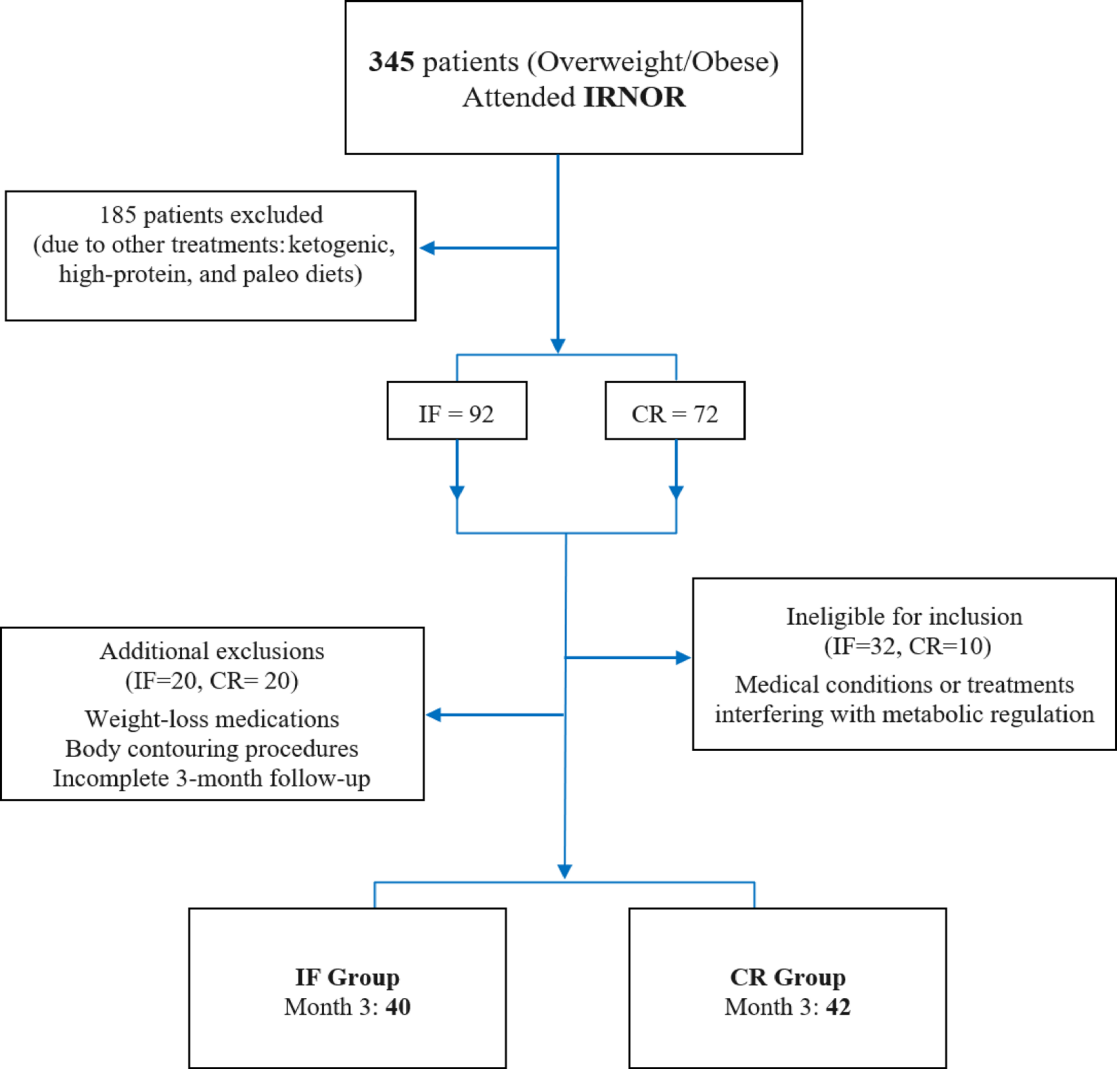
Flow diagram illustrating the analysis of study subject subgroups. IF: intermittent fasting; CR: calorie restriction.

From July to October 2023, 345 overweight or obese individuals were enrolled in the study at the IRNOR center, where they received treatment. The IRNOR protocol was applied consistently to all participants to enhance the reliability of data collection. Figure 1 offers additional details on the participant population and the screening process used in the study. A total of 82 participants with an average age of 35.55 ± 12.18 years (70.7% female) completed the three-month study, 40 in the 5:2 IF group and 42 in the CR group. Laboratory data at both the beginning and final stage of the study were available for 30 participants in each group.

Table 1. summarizes the demographic data, categorized by study groups. No significant difference was observed between the 5:2 IF and CR groups as for demographic characteristics including age, gender, education level, marital status, smoking habits, medical history, and energy intake (*P* > 0.05).

**Table 1:** Baseline characteristic of study participants

Variables	Groups		P-value
	IF (n=40)	CR (n=42)	
Age (year), median (IQR)	30 (25-44.75)	35 (28.75-44)	0.1
Gender, n (%)			
Male	12 (30.0)	12 (28.6)	0.89
Female	28 (70.0)	30 (71.4)	
Education, n (%)			
Diploma or under	16 (40.0)	14 (33.3)	0.53
University-educated	24 (60.0)	28 (66.7)	
Marital status, n (%)			
Married	27 (67.5)	24 (57.1)	0.44
Single	13 (32.5)	17 (40.5)	
Smoking, n (%)			
Yes	5 (12.5)	4 (9.5)	0.67
No	35 (87.5)	38 (90.5)	
History of chronic disease, n (%)			
Dyslipidemia	8 (20.0)	5 (11.9)	0.32
Hypertension	7 (17.5)	3 (7.1)	0.15
Medications, n (%)			
Lipid-lowering drugs	3 (7.5)	3 (7.1)	0.95
Pressure-lowering drugs	3 (7.5)	2 (4.8)	0.6
Energy (kcal), median (IQR)	1534.3 (714.35)	1986.8 (1127)	0.06

To compare groups, Mann-Whitney U test was used for continuous variables and chi-square test was performed for categorical variables. Abbreviations: IF: intermittent fasting; CR: calorie restriction.

During the three-month follow-up, SBP, MAP, and RPP rate decreased significantly in both groups (*P* < 0.05). At the end of week 12, SBP was significantly lower in the 5:2 IF group (123.78 ± 9.95 mmHg) compared to the CR group (127.62 ± 12.16 mmHg) (*P* = 0.02). While both DBP and pulse rate significantly decreased in the 5:2 IF group (DBP: from 89.93 ± 13.48 mmHg to 82.25 ± 9.75 mmHg, *P* < 0.001; pulse rate: from 89.93 ± 13.85 beats/minute to 84.50 ± 14.51 beats/minute, *P* = 0.01), no significant difference was observed between the 5:2 IF and CR groups at the end of the study (DBP: *P* = 0.49; PR: *P* = 0.34). Additionally, a significant difference was observed between the two groups in terms of pulse pressure at the end of the study (5:2 IF group: 41.53 ± 6.76 mmHg, CR group: 46.51 ± 9.36 mmHg, *P* = 0.03) (Table 2).

**Table 2:** Changes in blood pressure markers before and after the study in CR and IF groups.

Variables	Group	Baseline	Week 12	Change	*P*-value ^a^	*P*-value ^c^
Systolic BP (mmHg)	IF CR	134.63 ± 13.27 131.15 ± 15.02	123.78 ± 9.95 127.62 ± 12.16	−10.85 ± 12.23 −3.54 ± 9.21	< 0.001 0.02	0.02
Diastolic BP (mmHg)	IF CR	89.93 ± 13.48 83.26 ± 12.90	82.25 ± 9.75 81.10 ± 11.94	−7.68 ± 12.79 −2.15 ± 8.34	< 0.001 0.12	0.49
Pulse Rate (beats/minute)	IF CR	89.93 ± 13.85 88.59 ± 13.32	84.50 ± 14.51 85.56 ± 12.76	−5.43 ± 13.36 −3.03 ± 12.01	0.01 0.12	0.34
Mean Artery Pressure (mmHg)	IF CR	104.50 ± 12.74 98.90 ± 12.76	95.75 ± 9.36 96.26 ± 11.16	−8.75 ± 11.76 −2.41 ± 7.21	< 0.001 0.03	0.15
Pulse Pressure (mmHg)	IF CR	44.70 ± 9.30 47.90 ± 9.72	41.53 ± 6.76 46.51 ± 9.36	−3.18 ± 10.03 −1.38 ± 9.73	0.05 0.38	0.03
Rate Pressure Product	IF CR	12143.45 ± 2368.36 11694.15 ± 2641.30	10471.00 ± 2201.08 10944.05 ± 2094.83	−1672.45 ± 2131.76 −750.10 ± 1692.52	< 0.001 0.01	0.05

^a^ Paired sample T-test, ^c^ Analysis of Covariance (ANCOVA), adjusted for baseline values of each variable, age, sex and BMI. Abbreviations: IF: intermittent fasting; CR: calorie restriction; BP: blood pressure.

At week 12, a significant decrease in serum triglyceride levels was observed in the 5:2 IF group (from 145.59 ± 56.21 mg/dL to 124.96 ± 50.18 mg/dL, *p* = 0.04), while no significant difference was found between the two groups (*P* = 0.63). Cholesterol, LDL-C, and HDL-C levels remained unchanged within and between the two groups (*P* = 0.59, *P* = 0.84, and *P* = 0.42, respectively). Fasting plasma glucose and HbA1c levels also showed no significant changes in either the intra-group or inter-group comparisons (*P* = 0.67, and *P* = 0.15 respectively). While both ALT and AST significantly decreased in the 5:2 IF group (ALT: from 31.85 ± 24.11 IU/L to 25.07 ± 15.86 IU/L, *P* < 0.001; AST: from 26.18 ± 9.99 IU/L to 21.49 ± 8.68 IU/L, *P* < 0.001), no significant difference was observed between the two groups at the end of the study (ALT: *P* = 0.44; AST: *P* = 0.23). No significant changes were observed in urea, creatinine, and uric acid levels within or between the groups (*P* > 0.05) (Table 3).

**Table 3:** Changes in biochemistry tests before and after the study in CR and IF groups.

Variables	Group	Baseline	Week 12	Change	*P*-value	*P*-value ^c^
Triglyceride (mg/dL)	IF CR	145.59 ± 56.21 113.38 ± 40.90	124.96 ± 50.18 105.07 ± 44.14	−20.63 ± 49.67 −8.31 ± 36.36	0.04 ^a^ 0.11 ^a^	0.63
Cholesterol (mg/dL)	IF CR	180.17 ± 38.41 179.86 ± 31.11	171.57 ± 44.48 175.62 ± 40.89	−8.60 ± 25.82 −4.24 ± 21.54	0.08 ^a^ 0.30 ^a^	0.59
LDL-C (mg/dL)	IF CR	100.08 ± 31.22 112.30 ± 26.26	98.66 ± 27.95 107.03 ± 31.77	−1.42 ± 27.04 −5.27 ± 19.47	0.74 ^a^ 0.51 ^a^	0.84
HDL-C (mg/dL)	IF CR	42.14 ± 7.53 42.90 ± 8.95	41.73 ± 6.75 43.67 ± 10.63	−0.41 ± 7.01 0.77 ± 6.33	0.78 ^a^ 0.15 ^a^	0.42
FPG (mg/dL)	IF CR	93.92 ± 13.17 94.57 ± 12.34	89.33 ± 1.85 90.97 ± 11.83	−4.59 ± 13.51 −3.60 ± 10.10	0.06 ^a^ 0.06 ^a^	0.67
HbA1c (%)	IF CR	5.39 ± 0.72 5.42 ± 0.86	5.08 ± 0.40 5.34 ± 0.18	−0.31 ± 0.72 −0.08 ± 0.44	0.06 ^a^ 0.48 ^a^	0.15
ALT (IU/L)	IF CR	31.85 ± 24.11 27.21 ± 19.93	25.07 ± 15.86 23.77 ± 14.31	−8.13 ± 12.29 −3.17 ± 17.80	< 0.001 ^b^ 0.31 ^b^	0.44
AST (IU/L)	IF CR	26.18 ± 9.99 24.92 ± 11.63	21.49 ± 8.68 23.08 ± 9.09	−4.70 ± 7.89 −1.84 ± 8.33	< 0.001 ^a^ 0.28 ^a^	0.23
Urea (mmol/L)	IF CR	26.85 ± 4.38 25.67 ± 7.07	27.92 ± 8.60 22.78 ± 6.65	1.08 ± 6.76 −2.89 ± 4.96	0.58 ^a^ 0.12 ^a^	0.34
Creatinine (mg/dL)	IF CR	0.94 ± 0.18 0.90 ± 0.13	0.95 ± 0.17 0.87 ± 0.11	0.01 ± 0.17 −0.03 ± 0.17	0.66 ^a^ 0.48 ^a^	0.13
Uric acid (mg/dL)	IF CR	5.20 ± 1.32 5.21 ± 1.74	4.94 ± 1.32 4.76 ± 1.69	−0.26 ± 1.16 −0.45 ± 1.22	0.27 ^a^ 0.15 ^a^	0.26
TSH (µU/mL)	IF CR	3.53 ± 2.72 3.80 ± 3.39	3.62 ± 6.40 4.50 ± 6.48	0.26 ± 7.40 0.60 ± 6.54	0.28 ^b^ 0.79 ^b^	0.65

^a^ Paired sample T-test, ^b^ Wilcoxon signed rank test, ^c^ Analysis of Covariance (ANCOVA), adjusted for baseline values of each variable, age, sex and BMI. Abbreviations: IF: intermittent fasting; CR: calorie restriction; LDL-C: low density lipoprotein-cholesterol; HDL-C: high density lipoprotein-cholesterol; FPG: fasting plasma glucose; HbA1c: hemoglobin A1c; ALT: alanine transaminase; AST: aspartate aminotransferase; TSH: thyroid stimulating hormone.

**Fig. 2:**
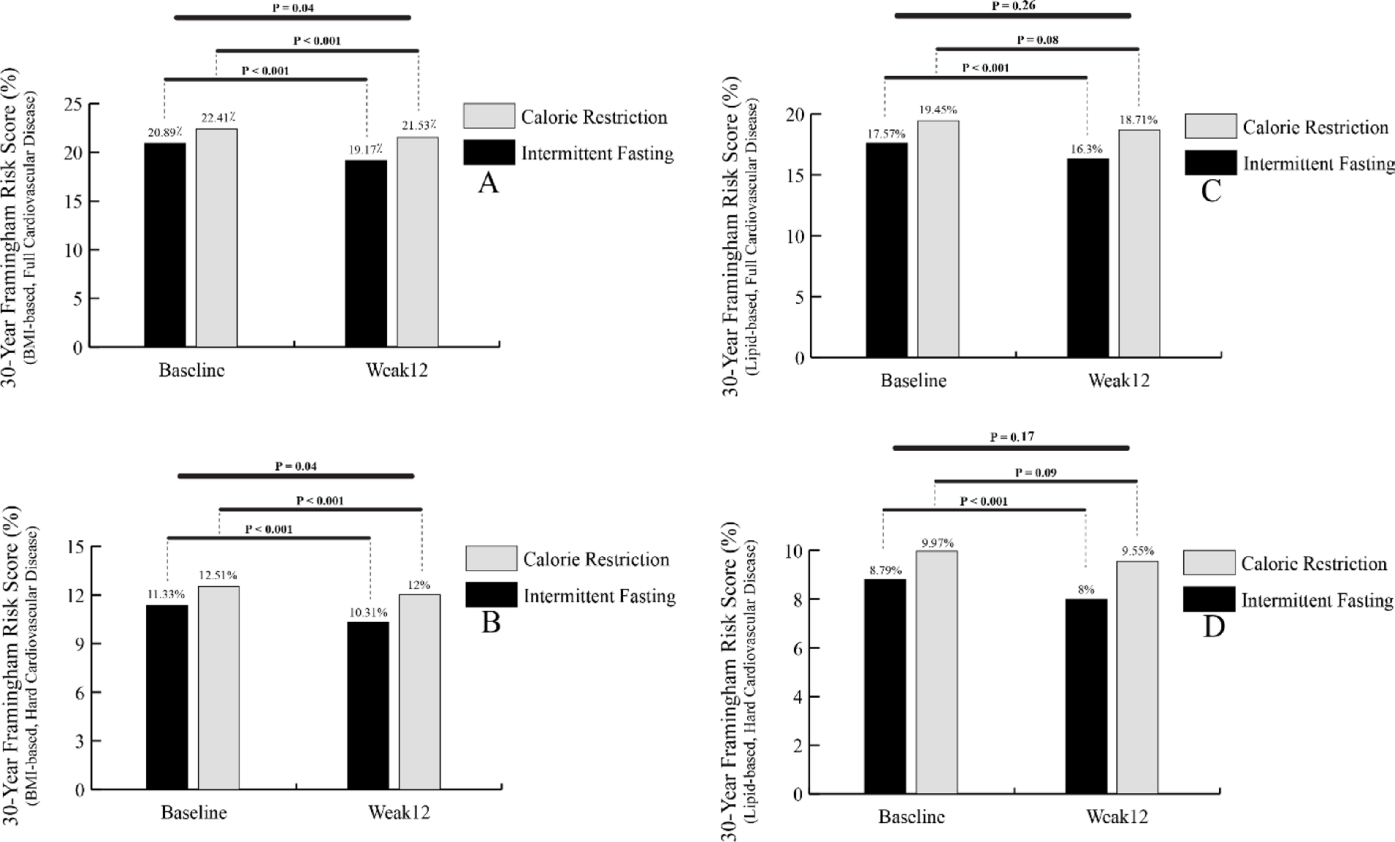
Changes in 30-year cardiovascular risk scores before and after the study in different study groups.

There were no significant changes observed for non-HDL cholesterol, coronary risk index, triglyceride-to-HDL ratio, atherogenic index, and atherogenic index of plasma in either intra-group and inter-groups comparisons (*P* > 0.05). As shown in Fig. 2A and B, both full and hard cardiovascular diseases showed significant decreases in 30-year Framingham risk score (FRS) (based on BMI) in the IF and CR groups (*P* < 0.001). At the end of week 12, the FRS for full CVD (BMI-based) was significantly lower in the 5:2 IF group (19.17 ± 14.13) compared to the CR group (21.53 ± 15.10) (*P* = 0.04). Similarly, for hard CVD (BMI-based), the score was significantly lower in the 5:2 IF group (10.31 ± 8.51) compared to the CR group (12.00 ± 9.88) (*P* = 0.04).

In the 5:2 IF group, 30-year FRS (based on lipids) for both full and hard cardiovascular diseases showed significant decreases (*P* < 0.001). The CR group did not demonstrate significant reductions in FRS (based on lipids) (full CVD: *P* = 0.08, hard CVD: *P* = 0.09), and no significant differences were found between the groups (full CVD: *P* = 0.26, hard CVD: *P* = 0.17) (Fig. 2C and D) (Table 4). There were no statistically significant differences in physical activity level, or in the intake of energy, carbohydrates, fats, or proteins either within groups or between the two groups (*P* > 0.05) (Table 5).

**Table 4:** Changes in cardiovascular risk scores before and after the study in CR and IF groups ^a^ paired sample T-test, ^b^ Wilcoxon signed rank test, ^c^ analysis of covariance (ANCOVA), adjusted for baseline values of each variable, age, sex and BMI. Abbreviations: IF: intermittent fasting; CR: calorie restriction; TG: triglyceride; HDL-C: high density lipoprotein-cholesterol; BMI: body mass index; FRS: Framingham risk score.

Variables	Group	Baseline	Week 12	Change	*P*-value	*P*-value ^c^
Non-HDL	IF CR	137.85 ± 36.39 136.72 ± 29.66	130.06 ± 42.93 131.72 ± 37.29	−7.79 ± 23.77 −5.00 ± 19.79	0.08 ^a^ 0.18 ^a^	0.75
Coronary risk index	IF CR	4.34 ± 1.06 4.27 ± 0.88	4.21 ± 1.19 4.10 ± 0.91	−0.14 ± 0.69 −0.17 ± 0.56	0.29 ^a^ 0.11 ^a^	0.73
TG: HDL ratio	IF CR	3.62 ± 1.73 2.79 ± 1.27	3.15 ± 1.37 2.52 ± 1.18	−0.47 ± 1.39 −0.27 ± 1.00	0.29 ^b^ 0.12 ^b^	0.38
Atherogenic index	IF CR	2.40 ± 0.74 2.71 ± 0.76	2.44 ± 0.84 2.52 ± 0.76	0.04 ± 0.66 −0.18 ± 0.49	0.75 ^a^ 0.05 ^a^	0.30
Atherogenic index of plasma	IF CR	0.52 ± 0.18 0.40 ± 0.20	0.46 ± 0.21 0.36 ± 0.20	−0.07 ± 0.17 −0.05 ± 0.16	0.06 ^a^ 0.12 ^a^	0.58
30y-FRS (%) (BMI-based, Full CVD)	IF CR	20.89 ± 14.74 22.41 ± 15.30	19.17 ± 14.13 21.53 ± 15.10	−1.71 ± 2.79 −0.88 ± 2.12	< 0.001 ^b^ < 0.001 ^b^	0.04
30y-FRS (%) (BMI-based, Hard CVD)	IF CR	11.33 ± 9.14 12.51 ± 10.03	10.31 ± 8.51 12.00 ± 9.88	−1.01 ± 1.94 −0.51 ± 1.35	< 0.001 ^b^ < 0.001 ^b^	0.04
30y-FRS (%) (Lipid-based, Full CVD)	IF CR	17.57 ± 12.41 19.45 ± 14.10	16.30 ± 12.08 18.71 ± 13.57	−1.27 ± 2.94 −0.74 ± 2.70	< 0.001 ^b^ 0.08 ^b^	0.26
30y-FRS (%) (Lipid-based, Hard CVD)	IF CR	8.79 ± 6.60 9.97 ± 8.31	8.00 ± 6.32 9.55 ± 8.16	−0.79 ± 1.86 −0.41 ± 1.68	< 0.001 ^b^ 0.09 ^b^	0.17

**Table 5:** Changes in dietary intake and physical activity before and after the study in CR and IF groups.

Variables	Group	Baseline	Week 12	*P*-value	*P*-value ^c^
Physical activity, (MET-minute/week), mean ± SD	IF CR	810.74 ± 1048.43 712.89 ± 937.64	704.34 ± 817.05 613.81 ± 597.11	0.98 ^b^ 0.75 ^b^	0.71
Energy, (kcal/day), mean ± SD	IF CR	1754.50 ± 843.94 1991.40 ± 769.46	1248.27 ± 442.93 1466.07 ± 489.96	< 0.001 ^b^ < 0.001 ^a^	0.08
Carbohydrate, (gr/day), mean ± SD	IF CR	226.06 ± 116.41 277.37 ± 144.50	155.19 ± 63.08 190.85 ± 79.58	< 0.001 ^b^ < 0.001 ^b^	0.08
Fat, (gr/day), mean ± SD	IF CR	64.16 ± 51.32 71.77 ± 43.20	46.23 ± 25.79 51.41 ± 31.16	0.03 ^b^ < 0.001 ^b^	0.49
Protein, (gr/kg), mean ± SD	IF CR	0.86 ± 0.30 0.77 ± 0.28	0.88 ± 0.44 0.71 ± 0.28	0.74 ^a^ 0.25^a^	0.08

^a^ Paired T-test, ^b^ Wilcoxon signed rank test, ^c^ Analysis of Covariance (ANCOVA), adjusted for baseline values of each variable. Abbreviations: IF: intermittent fasting; CR: calorie restriction.

Comprehensive ANCOVA findings, including adjusted mean differences, standard errors, and 95% confidence intervals for all outcomes, are presented in Supplementary Tables S1-S3. Among the evaluated outcomes, significant between-group differences were observed for systolic blood pressure (B = −4.80 mmHg; 95% CI, −8.64 to −0.95; *P* = 0.02), pulse pressure (B = −3.93 mmHg; 95% CI, −7.44 to −0.42; *P* = 0.03), and the 30-year Framingham cardiovascular risk (BMI-based; full CVD: B = −0.84; 95% CI, −1.64 to −0.03; *P* = 0.04; hard CVD: B = −0.53; 95% CI, −1.06 to −0.01; *P* = 0.04).

## Discussion

This study compared the effects of 5:2 IF and CR on cardiovascular health markers. The study showed that both 5:2 IF and the CR dietary approaches were associated with favorable changes in some cardiometabolic risk factors over the 12-week study period. Both groups showed a decrease in systolic pressure, mean arterial pressure, and rate-pressure product. However, the IF group showed comparatively lower systolic blood pressure, pulse pressure, and BMI-based Framingham 30-year cardiovascular disease risk scores at the end of the study. However, no significant within-group or between-group differences were found in fasting glucose, HbA1c, total cholesterol, LDL-C, or HDL-C.

Previous research by Sutton et al. and Varady et al. supported these findings, as both studies reported reductions in SBP with IF^30,31^. Varady et al. also found no significant changes in DBP, while Sutton et al. reported reductions in both SBP and DBP after only 5 weeks of intermittent fasting^30,31^. A meta-analysis of 5:2 intermittent fasting studies showed similar reductions in SBP and DBP compared to those achieved through calorie restriction^10^, indicating that weight loss may be associated with these improvements. However, these results seem to contradict those of Antoni et al., who observed a greater decrease in SBP in the IF group compared to the CR group after 60 weeks^32^. The inconsistencies in the findings of these studies could be because of the variations in the study duration, study populations, measurement methods, timing of assessments, and levels of sodium intake.

This study presents novel findings by observing significant reductions in pulse rate for the IF group, while also highlighting the potential role of pulse pressure in cardiovascular health, an aspect that has received less attention in previous literature. Previous evidence suggested potential involvement of autonomic modulation and brain-derived neurotrophic factor (BDNF)^33^. BDNF may be associated with reductions in pro-inflammatory cytokines such as tumor necrosis factor-alpha (TNF-α), interleukin-1 beta (IL-1β), interleukin-6 (IL-6), and interleukin-8 (IL-8), which are implicated in the development of atherosclerosis^34^. Moreover, low-grade chronic inflammation, influenced by metabolic stress and adiposity, may provide a unifying pathway that connects intermittent fasting with vascular, neuroendocrine, and metabolic improvements^35^. Recent evidence also indicated that intermittent fasting may suppress activation of the nucleotide-binding domain-like receptor protein 3 (NLRP3) inflammasome, a key regulator of innate immune responses implicated in chronic inflammation and atherosclerosis^36^.

An important factor when interpreting the blood pressure reductions observed in this study concerns potential changes in sodium or fluid intake. Although daily sodium intake was not directly recorded under the IRNOR protocol, several methodological considerations indicated that these reductions were unlikely to result solely from temporary sodium or water loss. First, participants in both groups were instructed to maintain adequate hydration (at least 2 L/day) and were allowed to consume non-caloric beverages such as water, black coffee, and unsweetened tea on fasting days, minimizing the risk of dehydration. Second, the IF and CR meal plans were designed by the same clinical nutritionist and followed comparable dietary guidelines that emphasized moderate salt use without imposing sodium restriction, reducing the possibility of systematic between-group differences in sodium exposure. Third, biochemical indicators of renal function and hydration status (urea, creatinine, uric acid) remained stable throughout the intervention, suggesting no significant alterations in sodium or fluid balance. Moreover, all measurements were carried out under standardized pre-test conditions to minimize the possible impact of acute fluid shifts on body composition and blood pressure results.

Our study revealed a significant decrease in triglyceride levels in the IF group, while no significant change was observed in the CR group. Furthermore, there were no significant differences in triglyceride, total cholesterol, LDL-C, and HDL-C between the two groups. Previous research in this area has shown inconsistent results. For instance, Meng et al. reported reductions in total cholesterol and LDL-C, but no changes in triglyceride levels in their IF group^37^. Conversely, other studies have observed decreases in triglyceride levels with intermittent fasting^38,39^. These conflicting findings may be related to various factors, such as daily calorie intake, fasting duration, and initial triglyceride levels. Individual differences in these factors could impact the magnitude of changes in lipid profiles following intermittent fasting.

Despite the changes in triglycerides, HDL-C levels did not significantly change in either group. This is consistent with previous studies, indicating that short-term dietary interventions typically do not elevate HDL-C unless accompanied by longer duration or additional lifestyle modifications such as physical activity improvement. Several mechanisms have been proposed that may help explain the associations between intermittent fasting and lipid regulation. One proposed mechanism involves the reduced production of very low-density lipoprotein-cholesterol (VLDL-C) and triglycerides in the liver, along with increased fatty acid oxidation. This, in turn, can result in increased expression of the transcription factors peroxisome proliferator-activated receptor-alpha (PPAR-α) and peroxisome proliferator-activated receptor-gamma (PPAR-γ), which stimulate fatty acid oxidation and promote the production of apolipoprotein A (Apo A) while reducing the synthesis of apolipoprotein B (Apo B). The net effect of these changes is believed to be associated with lower levels of LDL-C and VLDL-C in the body, which could contribute to improved cardiovascular health^40^. Furthermore, intermittent fasting has been shown to increase the production of ketones in the body, even without weight loss, leading to potential health benefits and improved cellular resistance to diseases^41^.

The present study observed no significant differences in fasting plasma glucose and HbA1c levels in both groups. Consistent with these findings, other studies on non-diabetic individuals have also reported no significant changes in fasting blood glucose levels^42–45^. Sundfor et al., found that after 48 weeks of 5:2 IF, HbA1c levels decreased significantly from 6.5% to 5.3%, but this decrease was not significantly different from that of the daily CR dietary approach^45^. In a separate study on individuals with type 2 diabetes, it was found that the 5:2 IF was associated with greater reduction in HbA1c levels compared to continuous CR^46^. This absence of significant changes in glycemic markers may be explained by the inclusion of participants without diagnosed diabetes, yet possibly with varying degrees of glucose regulation.

The current study showed a statistically significant reduction in the 30-year Framingham risk score (BMI-based) in both groups, with the IF group being associated with a significantly lower score at the end of the study. Lipid-based scores also declined significantly in the IF group, though between-group differences were not significant. To the best of our knowledge, this is one of the first studies to assess the effect of 5:2 IF on CVD risk scores in overweight or obese subjects, highlighting the necessity for further research in this area. While this study is pioneering in its examination of the impacts of the 5:2 IF on CVD risk scores, other studies have explored the impact of various intermittent fasting strategies on CVD risk^47,48^. Nemati et al., for instance, discovered a significant reduction in the 10-year Framingham risk score following TRF^47^, while Schroeder et al. observed a 12% decrease in the 30-year CVD risk after 12 weeks of TRF^48^. From a methodological perspective, it is also important to note that metabolic profile variables were defined as the primary outcomes of the study, while CVD risk scores were secondary outcomes; hence, these findings should be interpreted with caution.

Several mechanisms may account for the benefits of IF on cardiovascular health. These include the regulation of circadian rhythm, potential reduction in oxidative stress, and induction of ketosis^34^. Fasting may help to synchronize body functions with the circadian cycle, preventing the negative effects of late-night eating, such as insulin resistance and disrupted sleep. Additionally, fasting reduces oxidative stress and inflammation by curbing the production of free radicals in mitochondria, thereby protecting the body from their damaging effects^49,50^. Furthermore, IF promotes a ketogenic state, which shifts the body’s main energy source from glucose to fat. This metabolic switch, known as ketosis, has been demonstrated to improve overall metabolic health, potentially explaining some of the observed benefits of IF^34^.

Although the reductions in systolic blood pressure, pulse pressure, and Framingham risk score found in this study were statistically significant, it is important to recognize that the absolute differences between the groups (around 3–5 mmHg for SBP and PP, and 1–2 points for the risk score) were relatively modest and may not reach the minimal clinically important difference, typically estimated at about 10 mmHg for systolic blood pressure^51^. Even so, the within-group analysis indicated that participants following the 5:2 IF regimen showed an average SBP reduction of nearly 11 mmHg, meeting this threshold and suggesting that the approach could offer meaningful benefits for individuals who adhere to it. From a broader perspective, even small reductions of 2–5 mmHg in the blood pressure of an average population have been associated with noticeable declines in stroke and coronary heart disease rates^52^. Therefore, while the changes observed here may seem modest, they could still have important implications.

This real-world study (RWS) provides valuable insight into the associations between weight loss strategies and cardiovascular outcomes by reflecting practical settings, thus enhancing our understanding of this dietary approach. The 12-week period was chosen to allow enough time for observing meaningful short-term changes in cardiovascular risk factors, including blood pressure and lipid profile, which typically respond quickly to dietary changes^53^. This duration also supported better feasibility and adherence among participants, making it appropriate for real-world clinical settings^54^. The similarity in baseline characteristics between groups helped reduce potential bias. Examining comprehensive cardiovascular markers like pulse pressure and pulse rate alongside the 5:2 IF adds novelty to the research, especially considering its registry-based design. While the findings are promising, the study has inherent limitations associated with observational designs. First of all, participant blinding was not feasible, since individuals were aware of their dietary pattern as in the case of instructing the IF group to fast two days per week. Moreover, although the standardized approach helped minimize variability, it was not possible to control for all potential confounding factors, such as motivation, socioeconomic status, and health literacy. Therefore, some residual confounding and selection bias cannot be fully excluded. Furthermore, self-reported dietary intake might have underestimated actual values or differed from them, leading to potential inaccuracies in the analysis.

The inherent ethnic and sociodemographic diversity within the Iranian population provides valuable insights into the applicability of these dietary approaches, even within a single national setting. Nevertheless, future studies involving broader or international populations are recommended to examine the sustainability of these findings in other populations.

## Conclusions

The present study suggests that IF, particularly the 5:2 IF, may be associated with greater improvement in systolic blood pressure and pulse pressure, as well as with lower 30-year cardiovascular risk score after three months. Although the magnitude of these improvements was modest and was observed over a 12-week period, this duration is long enough to allow for the detection of early cardiometabolic responses to dietary approaches. Further research with longer follow-up is suggested to confirm and extend these observations.

## Supplementary Information

Below is the link to the electronic supplementary material.

Supplementary Material 1

## Data Availability

The datasets generated and/or analyzed during the current study are not publicly available due to university data ownership policies, but are available from the corresponding author on reasonable request.
