# Supplementary material for: Comparative effects of intermittent fasting and calorie restriction on cardiovascular health in adults with overweight or obesity
Source: Sci Rep. 2025 Dec 15;16:2867. doi: 10.1038/s41598-025-32673-9 (PMC12827324; doi:10.1038/s41598-025-32673-9)
Supplement: Supplementary file 1 — Supplementary Material 1 [file 41598_2025_32673_MOESM1_ESM.docx]

**Supplementary Table S1. Adjusted mean differences (95% CI) for blood pressure indices between study groups**

| Variables | Adjusted mean difference (B) | SE | 95% CI  (Lower, Upper) | P-value |
| --- | --- | --- | --- | --- |
| Systolic BP (mmHg) | -4.80 | 1.93 | (-8.639, -0.95) | 0.02 |
| Diastolic BP (mmHg) | -1.39 | 1.99 | (-5.35, 2.58) | 0.49 |
| Pulse Rate (beats/minute) | -2.48 | 2.59 | (-7.64, 2.68) | 0.34 |
| Mean Artery Pressure (mmHg) | -2.61 | 1.78 | (-6.16, 0.95) | 0.15 |
| Pulse Pressure (mmHg) | -3.93 | 1.76 | (-7.44, -0.42) | 0.03 |
| Rate Pressure Product | -749.68 | 373.04 | (-1493.13, -6.22) | 0.05 |

Data are presented as adjusted mean difference (B), standard error (SE), and 95% confidence interval (CI) derived from ANCOVA, controlling for baseline values, age, sex, and BMI. Abbreviations: IF: intermittent fasting; CR: calorie restriction.

**Supplementary Table S2. Adjusted mean differences (95% CI) for biochemical parameters between study groups**

| Variables | Adjusted mean difference (B) | SE | 95% CI  (Lower, Upper) | P-value |
| --- | --- | --- | --- | --- |
| Triglyceride (mg/dL) | 5.11 | 10.66 | (-16.29, 26.52) | 0.63 |
| Cholesterol (mg/dL) | -3.31 | 6.07 | (-15.49, 8.86) | 0.59 |
| LDL-C (mg/dL) | 1.20 | 5.85 | (-10.52, 12.93) | 0.84 |
| HDL-C (mg/dL) | -1.32 | 1.62 | (-4.55, 1.91) | 0.42 |
| FPG (mg/dL) | -1.01 | 2.36 | (-5.74, 3.72) | 0.67 |
| HbA1c (%) | -0.22 | 0.15 | (-0.52, 0.08) | 0.15 |
| ALT (IU/L) | -2.03 | 2.61 | (-7.28, 3.22) | 0.44 |
| AST (IU/L) | -2.19 | 1.80 | (-5.80, 1.42) | 0.23 |
| Urea (mmol/L) | 2.53 | 2.56 | (-2.89, 7.95) | 0.34 |
| Creatinine (mg/dL) | 0.06 | 0.04 | (-0.02, 0.13) | 0.13 |
| Uric acid (mg/dL) | 0.31 | 0.27 | (-0.24, 0.87) | 0.26 |
| TSH (µU/mL) | -0.92 | 2.01 | (-4.98, 3.15) | 0.65 |

Data are presented as adjusted mean difference (B), standard error (SE), and 95% confidence interval (CI) derived from ANCOVA, controlling for baseline values, age, sex, and BMI. Abbreviations: IF: intermittent fasting; CR: calorie restriction; LDL-C: low-density lipoprotein cholesterol; HDL-C: high-density lipoprotein cholesterol; FPG: fasting plasma glucose; HbA1c: hemoglobin A1c; ALT: alanine transaminase; AST: aspartate transaminase; TSH: thyroid-stimulating hormone.

| Variables | Adjusted mean difference (B) | SE | 95% CI  (Lower, Upper) | P-value |
| --- | --- | --- | --- | --- |
| Non-HDL | -1.81 | 5.60 | (-13.05, 9.43) | 0.75 |
| Coronary risk index | 0.06 | 0.17 | (-0.28, 0.39) | 0.73 |
| TG: HDL ratio | 0.25 | 0.28 | (-0.32, 0.81) | 0.38 |
| Atherogenic index | 0.16 | 0.15 | (-0.14, 0.46) | 0.30 |
| Atherogenic index of plasma | 0.03 | 0.05 | (-0.07, 0.12) | 0.58 |
| 30y-FRS (%)  (BMI-based, Full CVD) | -0.84 | 0.41 | (-1.64, -0.03) | 0.04 |
| 30y-FRS (%)  (BMI-based, Hard CVD) | -0.53 | 0.27 | (-1.06, -0.01) | 0.04 |
| 30y-FRS (%)  (Lipid-based, Full CVD) | -0.59 | 0.52 | (-1.63, 0.45) | 0.26 |
| 30y-FRS (%)  (Lipid-based, Hard CVD) | -0.45 | 0.33 | (-1.11, 0.20) | 0.17 |

**Supplementary Table S3. Adjusted mean differences (95% CI) for cardiovascular risk indices between study groups**

Data are presented as adjusted mean difference (B), standard error (SE), and 95% confidence interval (CI) obtained from ANCOVA, controlling for baseline values, age, sex, and BMI. Abbreviations: IF: intermittent fasting; CR: calorie restriction; FRS: Framingham risk score; CVD: cardiovascular disease; TG: triglyceride; HDL: high-density lipoprotein; BMI: body mass index.
